# Effect of Heat Transfer Medium and Rate on Freezing Characteristics, Color, and Cell Structure of Chestnut Kernels

**DOI:** 10.3390/foods12071409

**Published:** 2023-03-26

**Authors:** Lina Cheng, Weijun Wu, Jinghao Li, Xian Lin, Jing Wen, Jian Peng, Yuanshan Yu, Jieli Zhu, Gengsheng Xiao

**Affiliations:** 1 Sericultural & Argi-Food Research Institute, Guangdong Academy of Agricultural Sciences/Key Laboratory of Functional Foods, Ministry of Agriculture and Rural Affairs/Guangdong Key Laboratory of Agricultural Products Processing, No.133 Yiheng Street, Dongguanzhuang Road, Tianhe District, Guangzhou 510610, China; linake125@163.com (L.C.);; 2 College of Food Science and Technology, Zhongkai University of Agricultural and Engineering, Guangzhou 510631, China

**Keywords:** chestnut kernels, heat transfer medium, heat transfer rate, freezing characteristics, cell structure

## Abstract

This paper compared the effects of air and nitrogen on the freezing characteristics, color, and cell structures of chestnut kernels at different rates of heat transfer and adopted liquid nitrogen spray quick-freezing (NF_−40 °C/−60 °C/−80 °C/−100 °C_) and still air freezing (AF_−20 °C/−40 °C_) as the freezing methods. The ratio of heat transfer coefficients in N_2_ groups was two times as high as those in air groups, and NF_−100 °C_ and NF_−80 °C_ showed better freezing characteristics, good protection for cytoskeletons, and the color was similar to those of the fresh group. Taking both Multivariate Analysis of Variance (Principal Components Analysis and Cluster Analysis) and economic factors, NF_−80 °C_ can be used as a suitable method for chestnut kernel freezing. When the ambient freezing temperature was lower than *T_g_*, both NF and AF treatment groups presented poor quality. The rate and medium of heat transfer jointly influenced the freezing characteristics and quality. The former had a greater effect than the latter, however.

## 1. Introduction

Chestnut (Castanea mollissima Blume), belonging to the dicotyledon of Castanea of Fagaceae, is a kind of nut with rich nutrition, high medicinal value, and unique taste. It is praised as the “King of Nuts”, winning the love of consumers at home and abroad [1]. With a vast market, large quantity, and high yield, diseases, pests, water loss, and germination of the chestnuts easily occur in the storage and transportation of chestnuts. The current freshness-keeping technology of chestnuts mainly includes coating, heat, irradiation treatment, preservatives, etc. However, the storage period is still short, severely affecting the added value and reducing the economic benefits of the industry. How to prolong the storage period of chestnuts remains a major issue in the sustainable development of this industry.

Freezing is an effective method of food preservation and storage. The freezing method significantly impacts food freezing characteristics and further affects food microstructures. This impact can be reflected in the color, taste, and nutritional components [2,3]. The conventional freezing method has drawbacks, such as a low freezing rate and heat transfer coefficient, which lowers the quality of frozen products [4]. The heat transfer medium and ambient freezing temperature can be changed to enhance the heat transfer rate. Liquid nitrogen spray quick-freezing (NF), with N_2_ as the heat transfer medium, has advantages, such as a high freezing rate, more latent heat of vaporization [5], shorter time of passing through the maximum ice crystal generation zone, and uniform distribution of small ice crystals. This can maximize the retention of the original food taste of the frozen products [6,7]. According to research on blueberry freezing performed by Cheng et al. [8], NF is significantly better than the conventional freezing method in terms of freezing speed and nutrition characteristics. Additionally, NF is also the best freezing method to achieve the glass transition temperature (*T_g_*) of food [9], which plays an important role in keeping the original food quality [10]. In recent years, NF has been more widely adopted in the industrial food field. With its advanced equipment and lower cost, NF technology has begun to be widely used in fruits and vegetables [11,12,13], in addition to aquatic products with high economic benefits. There remains no related report on the application of NF in freezing fresh chestnut kernels. By adopting NF and conventional still air freezing at different ambient temperatures, this research compared the impact of the two heat transfer media (i.e., N_2_ and air) and the heat rate transfer on the freezing characteristics, color, and cellular structures of the chestnut kernels. It aimed to describe how the freezing characteristics affect the microstructure and further influence the appearance, quality, and components. The research results will enrich freezing theory and provide a technical reference for chestnut freezing and even the whole fruit and vegetable freezing business.

## 2. Materials and Methods

### 2.1. Sample Preparation

Chestnut kernels (Luotian “June Exposure” variety) were purchased from Luotian, Hubei Province, and transported in a cold chain to the laboratory. Chestnut kernels were selected with uniform size and shape, similar color, vacuum-packed in sealed bags (13 cm × 18 cm) with 6 kernels per bag, and then pre-cooled at 4 °C for 12 h in cold storage for later use.

Samples frozen by liquid nitrogen spraying freezing (NF) in a liquid nitrogen freezer (QF60-, Dejieli Refrigeration Technology Ltd., Shenzhen, China) or still air rapid freezing (AF) in a super air cooling machine blast freezer (HR05-SD2, Foshan Huaer Refrigeration Equipment Co., Ltd., Foshan, China) were denoted as NF_−40 °C_, NF_−60 °C_, NF_−80 °C_, NF_−100 °C_, AF_−20 °C_, and AF_−40 °C_ groups, respectively.

In order to illustrate the quality change caused by freezing, the frozen chestnut kernels were thawed at 4 °C for physical and chemical index analysis.

### 2.2. Regents

1,1-diphenyl-2-trinitrophenylhydrazine (DPPH), water-soluble vitamin C, water-soluble vitamin E (Trolox), phenol, methanol, anhydrous ethanol, folin phenol, concentrated hydrochloric acid, concentrated sulfuric acid, sodium dihydrogen phosphate, disodium hydrogen phosphate, sodium carbonate, and other reagents were purchased from Fuchen (Tianjin) Chemical Reagent Co., Ltd. and were all analysis grade.

### 2.3. Freezing Characterization

The total freezing time was determined by a thermocouple temperature collector (*T* type thermocouple, TC-08 collector, Omega Engineering Inc., Connecticut, CT, USA) to record the time required from the initial temperature (10 °C) to the final freezing temperature (−20 °C).

The freezing rate (*r*) and the time of maximum ice crystal formation zone (*T_i_*) can be calculated as [14]:

(1) $$r=\left(T_{0}-T_{f}\right)/T$$

(2) $$T_{i}=T_{c}-T_{if}$$

where *T*_0_ is the initial temperature of chestnut kernels 4 °C; *T_if_* is the initial crystallization temperature, *T_f_* is the freezing end temperature −20 °C; *T_c_* and *T_if_* are the freezing time for the core temperature of the chestnut kernels to reach the maximum ice crystal zone inflection point temperature and the initial freezing point, respectively.

### 2.4. Heat Transfer Characteristics

During the freezing process, in order to simplify the calculation, this paper assumed that the temperature difference between the surface of the chestnut kernels and the heat transfer medium in the freezer was constant, and the natural convection in a limited space, only the convective heat transfer coefficient (*h*) between the chestnut kernels and the heat transfer medium was calculated, and the formula is as follows:

(3) $$h=Nu\frac{\lambda }{l}$$

(4) $$Nu=0.061\times {\left(Gr\times \mathrm{Pr}\right)}^{1/3}$$

(5) $$Gr=\frac{ga\Delta tl^{3}}{v^{2}}$$

(6) $$Pr=\frac{\mu C_{p}}{k}$$

where *l* is the characteristic size of the chestnut; *λ* is the thermal conductivity of the chestnut; *Cp* is the specific heat capacity of the heat transfer medium; *μ* is the aerodynamic viscosity of the heat transfer; *a* is the coefficient of volume change (inverse of the absolute temperature); *g* is the acceleration of gravity; Δ*t* is the temperature difference (difference between the initial temperature of 4 °C and the treatment temperature).

According to the above formula, the ratio of the heat transfer coefficient between the treatments can be calculated as follows:

(7) $$d=\frac{{\left(a\Delta t\mu C_{p}\right)}_{i}}{{\left(a\Delta t\mu C_{p}\right)}_{j}}$$

where *i* and *j* represent the treatments; *i*: AF_−20 °C_; *j*: AF_−40 °C_, NF_−40~−100 °C_.

The *Cp*, *μ* of air, and nitrogen at different temperatures were checked from gas substance properties tables. The *d* value is shown in Table 1.

### 2.5. Weight Loss (WL) and Distribution of Water States

The masses of the chestnut kernels before (*m*_1_) and after (*m*_2_) thawing were recorded for *WL* determination. *WL* was calculated as follow:

(8) $$WL\%=\frac{m_{1}-m_{2}}{m_{1}}\times 100\%$$

The distribution of water states in the chestnut kernels was determined according to the method described by our previous study [8] using an LF-NMR (NMI-20, Shanghai Niumag Electronic Technology Co., Ltd., Shanghai, China).

### 2.6. Color Analysis

Color parameters (∆E) were obtained according to the method demonstrated by Tian et al. [11], using a colorimeter (Ultra Scan VIS model, Hunter Lab Inc., Reston, VA, USA).

### 2.7. Hardness Quality

Hardness was determined by the method described by Cheng et al. [8], using a texture analyzer (TA XT Plus, Stable Micro System, Ltd., Surrey, UK).

### 2.8. Nutritional Ingredient

Vitamin C: The determination of Vc was consistent with the method reported in our previous study [8] with a fluorescence spectrophotometer (Cary Eclipse, Varian Inc., Palo Alto, CA, USA).

Soluble sugar: Change in soluble sugar was extracted with the method of hot water extraction and tested with the method of phenol-sulfuric acid, reported by Yang et al. [15]. The final results were expressed in terms of glucose standard tune conversion equivalents.

Polyphenol: Change in polyphenol content was determined by the description of Yu et al. [16], using an ultraviolet spectrophotometer (UV 1800, Shimadzu, Kyoto, Japan). Calculation results were expressed in gallic acid equivalents.

DPPH radical scavenging rate: DPPH was determined using an enzymatic assay with specific reference to the method of Sokol-Letowska et al. [17], and was expressed as Trolox equivalents (mg/g). DPPH scavenging rate was calculated according to Equation (9).

(9) $$\mathrm{DPPH}/\%=\frac{\left(A_{1}-A_{0}\right)-\left(A_{i}-A_{j}\right)}{\left(A_{1}-A_{0}\right)}\times 100$$

### 2.9. Polyphenol Oxidase (PPO) Activity and Peroxidase (POD) Activity

PPO and POD activities of fresh and thawed chestnut kernel were determined at wavelengths of 410 and 470 nm, respectively, according to the method reported by Martynenko and Chen [18]. The calculation results were expressed as the relative residual enzyme activity of PPO/POD.

### 2.10. Microscopic Observations

The microstructure changes in chestnut kernels were evaluated by a previous study [8], using a SU8020 scanning electron microscope (Hitachi, Japan). The integrity and collapse of the cellular skeleton were analyzed comparatively.

### 2.11. Statistical Analysis

All experiments were repeated in parallel at least three times, taken under the same conditions, and the results were expressed as mean ± standard deviation (mean ± std). The experimental data were analyzed statistically by SPSS software (SPSS Version 24.0, SPSS Inc., Chicago, IL, USA) and plotted by origin 2018. A significant difference in each treatment was expressed as *p* < 0.05.

## 3. Results

### 3.1. Thermal Transition of Fresh Chestnut Kernels Containing Freezable Water

**Figure 1:**
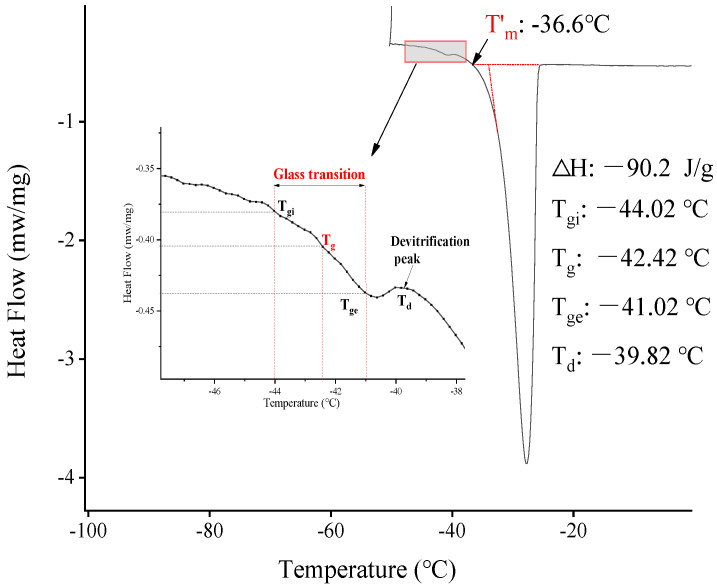
DSC thermogram of chestnut kernels containing freezable water (0.43 g water/g wet basis).

The differential scanning calorimetry (DSC) diagram of fresh chestnut kernels without annealing is shown in Figure 1, which belongs to the typical flow rate curve of the sample containing freezable water, including glass transition, and devitrification peak. The glass transition was the step phase of the curve and is enlarged in Figure 1. It is widely known that the glass transition temperature (*T_g_*) plays a crucial role in freezing food and maintaining its stability. *T_g_* depends primarily on the amount of water, as well as the composition and molecular weight of the solutes in the food [10]. The initial (*T_gi_*), mid (*T_g_*, −41.02 °C), and end-points (*T_ge_*) of the glass transitions of chestnut are shown in Figure 1. The onset of ice melting was found at Tm’ (−36 °C), which was also the end point of freezing, indicating all possible freezable water formed ice during cooling. The enthalpy of ice melting for chestnut containing freezable water (0.43 g water/g sample) was ∆H = −90.2 J/g. Furthermore, as rapid cooling led to partial freezing of the solution, the fluidity of the water molecules led to the recrystallization of the water in the amorphous matrix during the heating process. The devitrification temperature (*T_d_*) was observed at *T_d_* (−39.82 °C). These thermal transition characteristics of the chestnut kernels are similar to those of other fresh fruits, such as mango [19] and date syrup [20].

Table 1 shows the ratios of the heat transfer coefficient between the chestnut kernel surface and heat transfer media in different freezing treatment groups. The ratios of NF treatment groups with nitrogen as a medium were 1 to 2.4 times as high as those of AF treatment groups with air as a medium. In the same medium, the lower freezing temperature led to a higher heat transfer coefficient, which reflected the higher heat transfer rate. To simplify the calculation, the heat transfer between the still heat transfer medium and the material surface was considered. In the actual situation, the wind speed of liquid nitrogen spray quick-freezing was 1000 r/min, and the movement speed and heat transfer efficiency of the heat transfer medium in the case of NF were higher than those in the case of AF. NF and AF were both convective heat transfer, and their heat transfer rates mainly depended on the heat transfer coefficient, surface area, and temperature difference. Different treatment groups presented different temperature differences.

### 3.2. Freezing Characteristics

**Figure 2:**
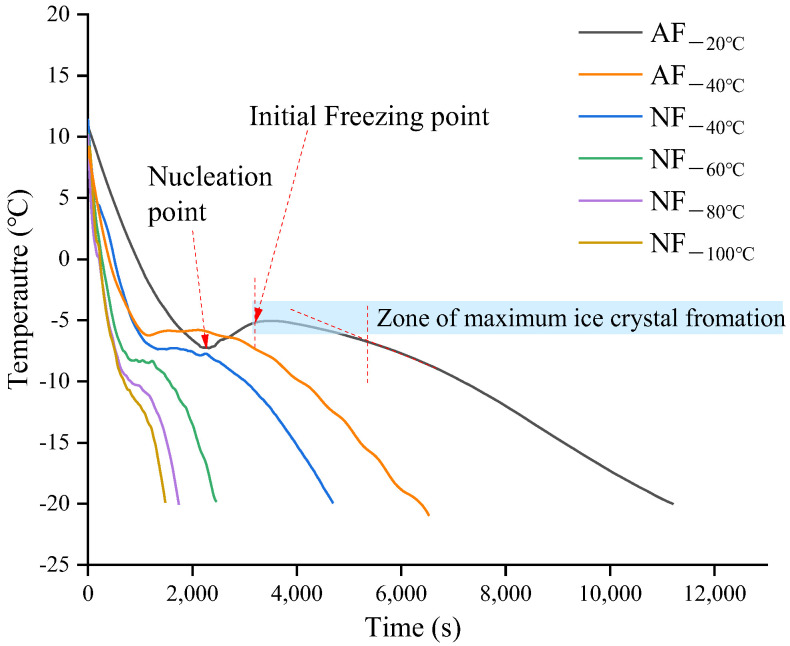
Freezing curve of chestnut kernels treated with different freezing methods.

Figure 2 shows the freezing curves of chestnut kernels under various freezing methods, similar to that of polar solution. This is mainly because food contains a large number of non-water matters [21]. The freezing process of chestnut kernels consisted of pre-cooling (rapid cooling), phase transition, and quenching. At the pre-cooling stage, rapid cooling occurred with a relatively steep freezing curve. At the phase transition stage, which refers to the maximum ice crystal generation zone, the free water was converted into ice with more heat to remove. At this stage, the temperature changed within only several degrees with a gentle slope of the curve, and it was also a relatively gentle section in the curve. In the AF_−20 °C_ treatment group, the curve at the phase transition stage was the gentlest and longest, while in the NF_−100 °C_ treatment group, the curve was the shortest. The reason is that the heat transfer coefficient of NF (100 W/m^2^K) was far larger than that of AF (5 W/m^2^K) [22]. The initial freezing point of chestnut kernels decreased with the decrease in freezing ambient temperature, which was mainly affected by the freezing rate and the initial characteristics of materials. During the freezing process, with the release of latent heat, the freezing point declined slowly, indicating that the freezing concentration effect occurred. The differences between NF and AF appeared mainly at the phase transition and quenching stages. It revealed that the differences in the quality change in frozen chestnut kernels were mainly caused by the differences in freezing rate and heat transfer temperature as well as the mechanical pressure suffered by ice crystals.

As is shown in Table 1, based on the total time of freezing (*T*) and the time of passing through the maximum ice crystal generation zone (*T_i_*), groups were ranked as follows: AF_−20 °C_ >> AF_−40 °C_ >> NF_−40 °C_ > NF_−60 °C_ > NF_−80 °C_ > NF_−100 °C_. *T_i_* values in NF treatment groups were even lower than those in AF treatment groups. In the NF_−40 °C_ and AF_−40 °C_ treatment groups with the same ambient temperature, the *T_i_* values of the former were 38% lower than those of the latter. This further suggests that the heat transfer performance of NF was significantly better than that of AF. When the temperature ranged from −40 °C to −100 °C, the lower ambient temperature for liquid nitrogen spray quick-freezing means lower *T* and *T_i_* values. This is consistent with the research conducted by Cheng et al. [8] on the liquid nitrogen spray quick-freezing of blueberries. In general, *T_i_* directly affected the size and distribution of ice crystals as well as the microstructures of cell tissues. A lower *T_i_* value needed a higher r value. The *T_i_* in NF_−100 °C_ was only about 270 s, equivalent to 1/8 of the *T_i_* value in the AF_−20 °C_ treatment group. This was consistent with the relationship between the two treatment groups in terms of average freezing rates. r values in the case of NF_−100 °C_ were the highest. The r value in the NF_−100 °C_ group was about seven times as high as that in the AF_−20 °C_ group. A similar trend existed in the research performed by Tian et al. [11] on the liquid nitrogen quick-freezing of cordyceps sinensis. In terms of the r value, NF was divided into two major categories: NF_−80 °C~−100 °C_ (r ≈ 1) and NF_−40 °C~−60 °C_ (r << 1). Higher r values meant a greater nucleation rate. The ice crystals were small and uniformly distributed in the food tissues, especially when the nucleation rate was higher than the growth rate of ice crystals. Their microstructures kept the initial state to a maximum extent after unfreezing. Therefore, from the perspective of heat transfer and freezing properties, the chestnut kernels treated with NF_−100 °C_ presented the best microstructure quality, followed by the NF_−80 °C_ treatment group with a freezing rate close to 1 °C/min.

### 3.3. Weight Loss and the Distribution of Water States

**Figure 3:**
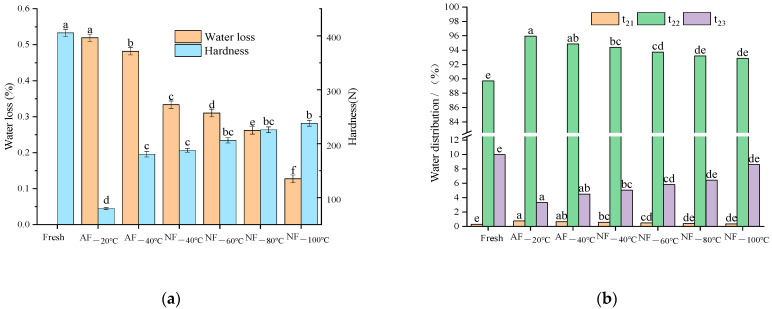
Effects of different freezing treatments on the (**a**) water loss and hardness; (**b**) water distribution of chestnut kernels. Note: In each figure, different letters mean significant differences (*p* < 0.05).

A higher heat transfer rate leads to a higher freezing rate and shorter freezing time, contributing to a lower juice loss rate after unfreezing [23]. As is shown in Figure 3a, the juice loss rate of the chestnut kernels in the NF_−40 °C_ group dropped by 30.81% compared with the AF_−40 °C_ group, and there was no remarkable difference in juice loss rate between the AF_−40 °C_ group and AF_−20 °C_ group. Moreover, NF_−40 °C_ was the crucial temperature to reduce the time of freezing chestnut kernels, and NF_−100 °C_ freezing was the critical temperature to protect the kernel quality. The juice loss rate decreased by 51.37% in NF_−100 °C_ compared with NF_−80 °C_. This indicates that liquid nitrogen quick-freezing is better than fridge freezing in terms of heat transfer and quality maintenance. According to the research performed by Lopkulkiaert et al. [24] on the quick-freezing of South American white shrimps, liquid nitrogen quick-freezing is significantly better than air-cooled and rack-contact freezing in terms of freezing rate and water holding capacity. *WL* was relatively low on the whole (0.12–0.52%). Because there was mainly non-flowing water in chestnut kernels with little free water, *T_2_*, the relaxation time of low-field NMR can indirectly reflect the freedom degree of water, the proportion of each peak area to the total area, and the content of different forms of water. *T*_21_*, T*_22_*,* and *T*_23_ indicated water changes in the plant tissue systems. *T*_21_ represents the bound water within the cell walls; *T*_22_ represents the low-mobility water in the intercellular space and cytoplasm; *T*_23_ represents free-flowing water with the greatest mobility in vacuoles [25]. According to Figure 3b, the proportion of free water in frozen–thawed chestnut kernels decreased (by 1.4–6.7%) remarkably, while the proportions of low-mobility water (by 3.14–6.2%) and bound water (by 0.06–0.46%) increased dramatically. There is a low content (roughly 43%) of water in fresh chestnut kernels. The ice crystals produced in the freezing process imposed mechanical and heat pressures, which altered the integrity of cellular tissues and the permeability of cell membranes, resulting in a loss of free water. The free water flowed to intercellular space and cytoplasm with only a little flowing to cell walls. The proportion of bound water slightly increased, mainly caused by a reduction in the total water content and a decrease in the proportion of free water. Overall, there was a smaller change in water content in NF treatment groups than that in AF treatment groups. The smallest water loss was in the NF_−100 °C_ group, followed by the NF_−80 °C_ group, and the AF_−20 °C_ group suffered the largest water loss. This was consistent with the changing trend in the juice loss rate. After freezing treatment in different conditions, the trends and differences in the changes in water content and distribution were the same as those of rates of heat transfer and freezing rates. It further reveals that heat transfer medium and rate are crucial factors for determining the juice loss of frozen–thawed products. In the freezing process, smaller ice crystals in the cells produced lower mechanical and heat transfer pressures, contributing to better maintenance of the integrity and permeability of cellular structures and greater protection from water.

### 3.4. Change in Color-Related Factors

**Figure 4:**
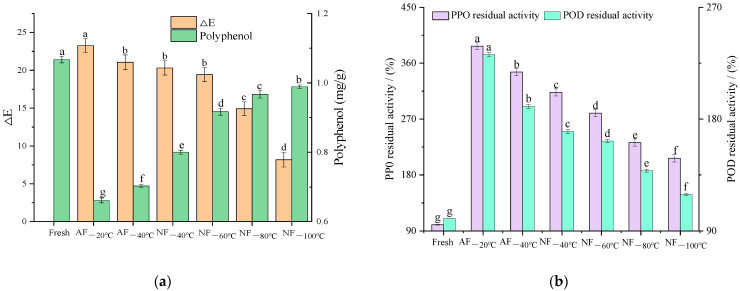
Effects of different freezing treatments on the (**a**) color and polyphenol content; (**b**) PPO and POD residual activity of chestnut kernels. Note: In each figure, different letters mean significant differences (*p* < 0.05).

As is shown in Figure 4a, after the freezing treatment with different heat transfer methods, the color of chestnut kernels darkened to different degrees. The color of chestnut kernels in the NF_−100 °C_ treatment group was closest to that of fresh chestnut kernels, and the color can be best protected. This was perhaps because different freezing methods formed different sizes and forms of ice crystals and contributed to the mechanical damage to chestnut kernels, the irreversible damage of cell walls, and the activation of oxidases, including polyphenol oxidase [8]. The activated enzymes had an enzyme-catalyzed reaction with phenolic compounds in the fruits. These oxidation products were converted into browned substances through an oxidative polymerization reaction [6]. The concentrations of oxygen and enzymes increased due to the relationship between phenols and aldehydes. This was also the key cause of the browning of fresh chestnut kernels after freezing.

According to Figure 4b, the residual enzyme activity of polyphenol oxidases (PPO) and peroxidases (POD) increased dramatically to different degrees. As the heat transfer and freezing rates rose, the enzymatic activation gradually decreased. Based on the residual enzyme activity, different treatment groups were ranked as follows: AF_−20 °C_ > AF_−40 °C_ > NF_−40 °C_ > NF_−60 °C_ > NF_−80 °C_ > NF_−100 °C_. It is noteworthy that in these treatment groups, enzymes were activated best in the AF_−20 °C_ treatment group. NF_−40 °C_ was the first crucial temperature for reducing the residual enzyme activity. The residual enzyme activities of PPO and POD in this group decreased by 65% and 60%, respectively, compared with those in the AF_−20 °C_ group. There was a significant difference between the NF_−80 °C_ group and the NF_−40 °C_ group in terms of residual enzyme activities of PPO and POD. Specifically, the residual enzyme activities of PPO and POD in the NF_−80 °C_ treatment group declined by 80% and 32%, respectively, indicating that −80 °C was the second crucial temperature in liquid nitrogen freezing. The residual enzyme activities of PPO and POD in the NF_−100 °C_ treatment group dropped by 19% and 22%, compared with those in the NF_−80_ treatment group. Therefore, according to the results mentioned above, the degree of enzymatic activation in the treatment groups with N_2_ as the heat transfer medium was significantly lower than that in the treatment groups with air as the heat transfer medium. For the same heat transfer medium, the higher the heat transfer rate, the lower the degree of enzymatic activation. This may be because the higher freezing rate led to smaller ice crystals and less damage to the cells. This caused the enzymes to locate within the initial cellular zones and avoid contact with substrates. It also better protected the high-order structures of enzymes, had less influence on the activity center of enzymes, and resulted in the activity being closer to the initial state. In addition, when the cellular structures of the kernels suffered greater damage, the bound enzymes were released and shifted into free enzymes. Overall, this improved the activity of the enzymes and catalyzed the enzymatic reaction of phenolic substances [26]. These factors darkened the color of the kernels. After freezing at −25 °C, the PPO in Agaricus Bosporus still had high activity [27]. Cano et al. [28] found that after freezing treatment at −18 °C for 7 days, the PPO activity in papain extract increased four times. Figure 3c shows the change in polyphenol content in the chestnut kernels after the freezing–thawing treatment. The different treatment groups presented similar change trends in polyphenol content and color on the whole. The decreased activation of enzymes meant a weaker enzymatic browning reaction, and the color of the kernels was better protected accordingly.

### 3.5. Vc and Soluble Sugar Changes

**Figure 5:**
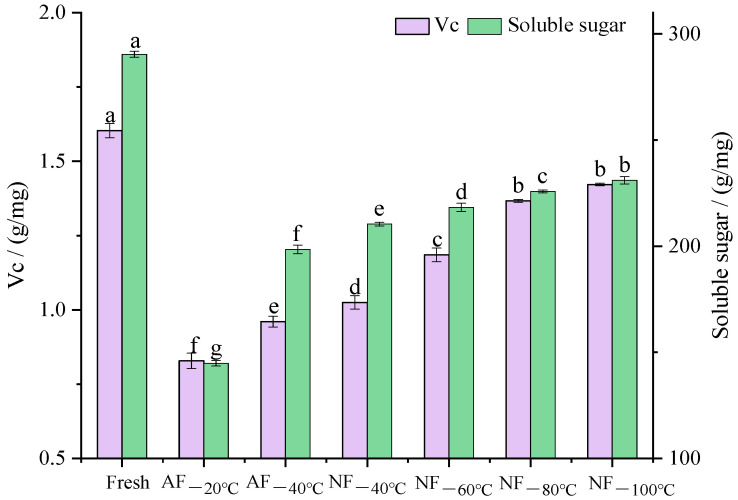
Effects of different freezing treatments on the Vc and soluble sugar of chestnut kernels. Note: In each column, different letters mean significant differences (*p* < 0.05).

Vc and soluble sugar, both soluble, are important functional nutritional components in chestnut kernels, which were closely associated with juice loss after freezing–thawing treatment. After NF and AF treatment with different heat transfer methods, the polysaccharides and Vc contents in chestnut kernels decreased. As is shown in Figure 5, according to the retaining rates of functional components, groups were ranked as follows: NF_−100 °C_ ≥ NF_−80 °C_ > NF_−60 °C_ > NF_−40 °C_ > AF_−40 °C_ > AF_−20 °C_. This changing trend was generally similar to that of the juice loss rate, and the retaining rate in the case of NF_−40 °C_ was 4% higher than that in the case of AF_−40 °C_. The above result indicated that nitrogen is a better medium for heat transfer than air. Based on further analysis, the retaining rate of Vc in the NF_−60 °C_ treatment group was 10% higher than that in the NF_−40 °C_ treatment group, and the retaining rate of Vc in the NF_−80 °C_ treatment group was 11% higher than that in the NF_−60 °C_ treatment group. After further reducing the temperature, the retaining rate of Vc in the NF_−100 °C_ treatment group was only 3% higher than that in the NF_−80 °C_ treatment group. In terms of the protection for polysaccharides, the retaining rates of soluble sugar in the NF_−60 °C_, NF_−80 °C_, and NF_−100 °C_ treatment groups were about 3%, 5%, and 7% higher, respectively, than the rates in the NF_−40 °C_ treatment group. The NF_−100 °C_ treatment group consumed more nitrogen than the NF_−80 °C_ treatment group. Considering the economic cost, NF_−80 °C_ treatment is considered a more suitable freezing method to protect the nutritional components in chestnut kernels. Moreover, the Vc content showed a greater decrease in all treatment groups than in polysaccharides. This is mainly due to the instability of Vc, which was decomposed in the freezing–thawing process.

### 3.6. Hardness and Microstructure Changes

The change trends of hardness (Figure 3a) of chestnut kernels in different treatment groups were similar to those of juice loss rate. However, the degree of hardness change (down by 41–80%) was far larger than that of the juice loss rate (down by 0.12–0.52%). This indicated that the hardness change is mainly determined by cytoskeleton change. The cellular microstructure is an important indicator to reveal the freezing effect. Freezing brought about inevitable and complex cell damage to some extent, and it may break the cell walls and cytomembranes and change the cell osmotic pressure. Because the freezing method and rate have a significant impact on the cellular structures [2], it is necessary to find a suitable freezing method to maintain the initial cellular structures best.

**Figure 6:**
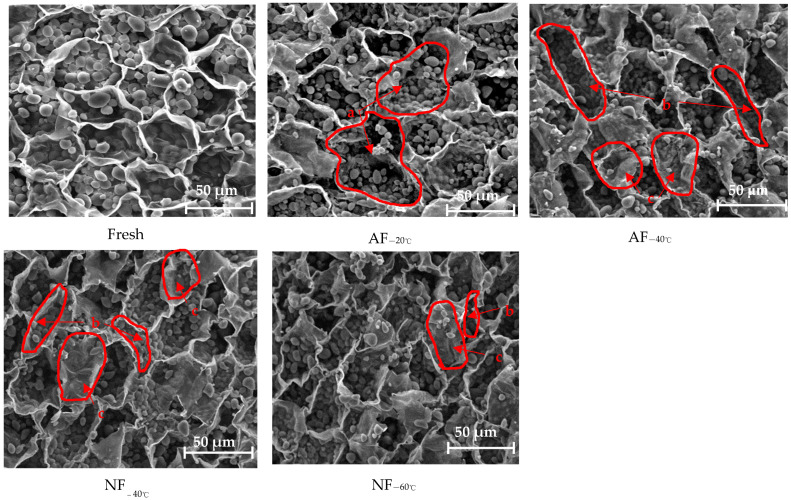

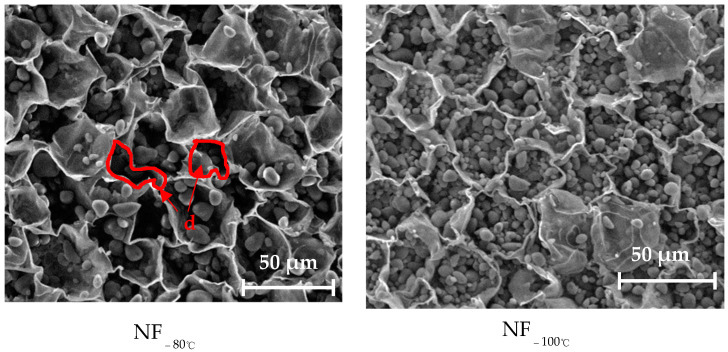
Microstructure of chestnut kernels treated by different freezing methods. a: collapse and puncture, b: cell gap, c: collapse, d: shrinkage. The SEM observation was carried out at a magnification of 200 times.

The tissue structures of sections of fresh chestnut kernels were plump and the cells were closely arranged, as is shown in Figure 6. There were obvious differences in cellular structures and arrangement between the quick-freezing groups and the slow-freezing groups after different treatment methods. The former can better maintain the cellular structures than the latter. According to the analysis of the Confocal Laser Scanning images of mangoes conducted by Charoenrein and Owcharoen [29], after quick-freezing treatment, the cell walls remained round and were similar to those of fresh mangoes, while the slow-freezing treatment led to significant changes in cellular structures with lower uniformness. In the AF_−20 °C_ treatment group with the lowest r value, the cells showed an obvious collapse with damaged structures in the cell walls and blurred cell boundaries. When the freezing temperature was lowered, long linear gaps appeared between holes in the AF_−40 °C_ treatment group. This was because the r value remained low, which indicated slow freezing and caused the water within cells to flow to intercellular space, forming large intercellular ice crystals [30] and increasing the intercellular space. NF_−40 °C_ showed narrower gaps between holes and a smaller collapse compared with AF_−40 °C_. When the treatment temperature dropped further, the cells at NF_−60 °C_ showed slight shrinkage and smaller intercellular space. The tissue images for the NF_−80 °C_ and NF_−100 °C_ groups showed an obvious improvement. This may be because a higher freezing rate reduced the time of passing through the maximum ice crystal generation zone. The small ice crystals uniformly distributed in the tissues protected the tissue structures. Therefore, the ambient temperature of −80 °C may be a key point for the liquid nitrogen spray quick-freezing treatment of chestnut kernels. In the NF_−100 °C_ group, the holes were denser, the tissue structures were plumper, and they were the most similar to fresh tissues. This was consistent with the change trends in related freezing characteristics and quality. This is similar to the results of the Ultrasound-Assisted Freezing research on potatoes conducted by Tian et al. [31]. In the treatment groups with the shortest time of passing through the maximum ice crystal generation zone, the SEM image showed denser cellular arrangement, while in the treatment groups with the longest time of passing through the maximum ice crystal generation zone, there existed cell collapse and shrinkage and poor quality of the frozen products. The microstructure changes in chestnut kernels were consistent with the heat transfer and freezing rates trends. It comprehensively proved that NF_−100 °C_ was the best treatment method to protect the microstructures of chestnut kernels, followed by NF_−80 °C_, which was acceptable for frozen product quality.

### 3.7. Multivariate Data Analysis

**Figure 7:**
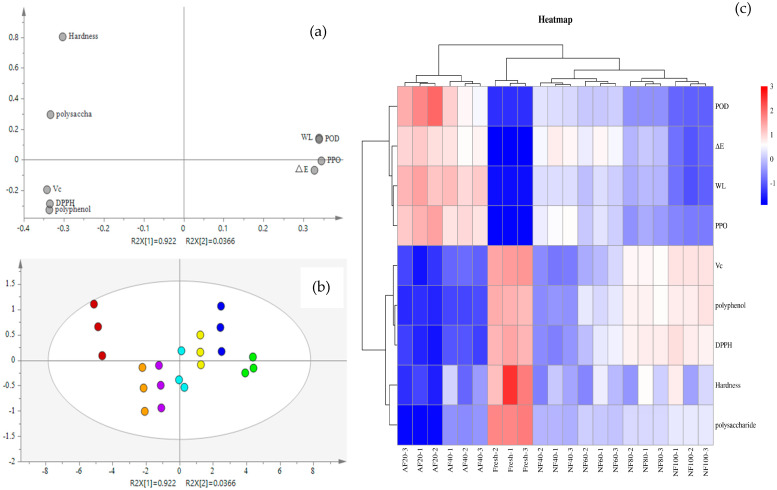
Loading scatter plot (**a**) and Score plot (**b**) of principal component analysis, and heatmap combined with the dendrogram of cluster analysis (**c**) were obtained based on the determined quality attributes. 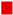 Fresh, 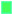 AF_−20 °C_, 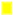 AF_−40 °C_, 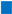 NF_−40 °C_, 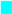 NF_−60 °C_, 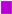 NF_−80 °C_, 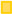 NF_−100 °C_.

Principal Component Analysis (PCA) is an unsupervised pattern recognition method that can show high-dimensional separation [32]. As is shown in the variable load diagram in Figure 7a, *WL*, ∆E, and PPO/POD were the principal components of PC1 that affected the quality change in chestnut kernels, and the contribution rate accounted for 92.2%. According to the PCA principle, groups with great similarity were clustered in the score plot. As is shown in Figure 7b, the untreated and treated groups were divided into four groups. NF_−100 °C_ and NF_−80 °C_ were within one group, and their quality indicators were closest to that of the fresh group. It should be noted that NF_−40 °C_, AF _−40 °C_, and AF_−20 °C_ showed positive scores in PC1. It indicates that the values of *WL*, ∆E, and PPO/POD in the three groups were high, and the quality of chestnut kernels was greatly damaged, which was consistent with the actual experimental data. This was mainly due to these three groups’ relatively low freezing rates, with freezing temperatures lower than *Tg*. The other groups, including the fresh group, obtained negative scores, indicating the high content of VC, polyphenols, and polysaccharides and a high hardness value in the chestnut kernels. Therefore, according to PCA scores, NF_−100 °C_ and NF_−80 °C_ are suitable treatment methods for freezing chestnut kernels.

To further verify the PCA results, Cluster Analysis was adopted. Regardless of sample categories, treatment groups with the most similar quality were clustered [33]. As is shown in Figure 7c, there were four major clusters, which was consistent with the PCA results. According to the above illustration, nitrogen is a more suitable heat transfer medium for freezing chestnut kernels than air. However, the ambient temperature of freezing had a more significant impact. That is, can the quality of frozen products be improved only when the freezing rate reaches a certain value.

## 4. Conclusions

The current study compared the influence of different heat transfer media (N_2_ vs. Air) and rates on freezing characteristics, color, and cytoskeletons of chestnut kernels. NF_−40 °C_, AF_−40 °C_, and AF_−20 °C_ treatment groups, whose ambient temperatures were lower than *T_g_*, showed lower freezing rates, leading to great change in color and great collapse of cytoskeletons of chestnut kernels. Nitrogen is, therefore, a more suitable heat transfer medium than air in terms of freezing chestnut kernels. Parameter changes in freezing characteristics significantly affected the color and cytoskeleton integrity. NF_−100 °C_ and NF_−80 °C_ showed higher heat transfer and freezing rates, which can better protect the quality of chestnut kernels. Taking the economic cost into consideration, it is recommended that NF_−80 °C_ should be a suitable method to freeze chestnut kernels. Meanwhile, this research further revealed that the heat transfer medium and rate are the crucial factors of freezing characteristics. They further affect the quality at the micro-level, nutrients, and appearance. This paper further illustrated the great potential of NF in preserving perishable foods and broadening its industrial application.

## Figures and Tables

**Table 1:** Technical index relating to heat transfer and freezing efficiency of chestnut kernels freezing.

Heat Transfer Medium	Freezing Temperature (°C)	*Cp* (kJ/kg K)	*μ* (10^−5^ pa∙s)	*d*	*T* (s)	*T_i_* (s)	*r* (°C/min)
Air	−20	1.0058	1.62383	1	11208 ± 113 ^f^	2182 ± 34 ^f^	0.165 ± 0.002 ^f^
	−40	1.0059	1.51872	1.60531	6531 ± 105 ^e^	1429 ± 30 ^e^	0.275 ± 0.004 ^e^
N_2_	−40	1.0421	1.46511	1.60437	4689 ± 110 ^d^	1063 ± 19 ^d^	0.385 ± 0.008 ^d^
	−60	1.0428	1.3613	2.03949	2457 ± 70 ^c^	642 ± 15 ^c^	0.72 ± 0.016 ^c^
	−80	1.0438	1.25383	2.328104	1740 ± 46 ^b^	403 ± 9 ^b^	0.993 ± 0.019 ^b^
	−100	1.0454	1.14238	2.48925	1480 ± 53 ^a^	270 ± 12 ^a^	1.159 ± 0.027 ^a^

Note: *t*, total freezing time; *T*, transit time of maximum ice crystal formation zone; *r*, freezing rate. In each column, different letters mean significant differences (*p* < 0.05).

## Data Availability

The data presented in this research are available on request from the corresponding author.
